# Facile Synthesis of Magnetic Biochar Derived from Burley Tobacco Stems towards Enhanced Cr(VI) Removal: Performance and Mechanism

**DOI:** 10.3390/nano12040678

**Published:** 2022-02-18

**Authors:** Baihui Cui, Zhihua Chen, Feihua Wang, Zihan Zhang, Yanran Dai, Dabin Guo, Wei Liang, Yu Liu

**Affiliations:** 1 Institute of Hydrobiology, Chinese Academy of Sciences, Wuhan 430072, China; cuibaihui0306@163.com (B.C.); wangfeihua93@ihb.ac.cn (F.W.); zihanzhang1018@gmail.com (Z.Z.); yanrandai@ihb.ac.cn (Y.D.); 2 Advanced Environmental Biotechnology Centre, Nanyang Environment & Water Research Institute, Nanyang Technological University, Singapore 637141, Singapore; guodabin2018@gmail.com (D.G.); CYliu@ntu.edu.sg (Y.L.); 3 School of Civil Engineering, Guangzhou University, Guangzhou 510006, China; 4 Key Laboratory for Yellow River and Huai River Water Environment and Pollution Control, School of Environment, Henan Normal University, Xinxiang 453007, China; chenzhihua@htu.edu.cn; 5 School of Civil and Environmental Engineering, Nanyang Technological University, Singapore 639798, Singapore

**Keywords:** magnetic biochar, Cr(VI) removal, Cr(III) immobilization, mechanism analysis

## Abstract

In this study, ferric-loaded magnetic burley tobacco stem biochar (MBTS) was synthesized via pyrolysis to improve the removal of Cr(VI). The results showed that MBTS had an adsorption capacity of 54.92 mg Cr(VI)/g, which was about 14 times higher than raw burley tobacco stem biochar (i.e., 3.84 mg/g). According to the findings obtained, a three-step mechanism of Cr(VI) removal by MBTS was further put forward, i.e., (1) Cr(VI) exchanged with hydroxyl groups on MBTS, (2) the reduction in Cr(VI) to Cr(III) mediated by oxygen-containing groups, and (3) the chelation of produced Cr(III) with the amino groups on MBTS. FTIR spectra further revealed that C-N, C-H, and C=C groups played an important role in Cr(VI) removal. Furthermore, the adsorption equilibrium and kinetics of Cr(VI) on MBTS could better be described by the Langmuir equation and pseudo-second-order rate equation. This study clearly demonstrated that ferric-loaded biochar derived from burley tobacco stems could serve as a cost-effective magnetic adsorbent for the high-efficiency removal of soluble Cr(VI) from wastewater. Tobacco stem-adsorbed Cr(VI) realized a green path for treating waste by waste.

## 1. Introduction

Heavy metals are assorted as one of a multitude of toxic materials in wastewater, which is of great significance to investigate its removal. Chromium (Cr) has been considered as one of the most hazardous wastewater metal pollutants due to its advanced mobility, solubility, and toxicity. Chromium (Cr) mainly exists in two patterns, i.e., hexavalent chromium (Cr(VI)) and trivalent chromium (Cr(III)), with excessive hazards caused by Cr(VI) than Cr(III) [1]. Cr(VI) exists widely in various industrial activities, such as metal electroplating, dyeing, tanneries, fertilizer, metallurgy, and fungicide production, and possesses a high solubility when entering into the aquatic environment [2]. It should be noted that it is indispensable to bring down the concentration of Cr(VI) as low as possibly reasonable before discharging. In view of this, various methods have been widely used for Cr(VI) removal, such as adsorption, biological degradation, chemical precipitation, reverse osmosis, ion exchange, and membrane separation [3]. Adsorption is largely applied among these, for its high-efficiency, environmentally friendly, operation-flexibility and low-cost [4,5].

Biochar is a pyrogenic black carbon acquiring increasing attention in recent years, which could be produced from biomass under oxygen-limited conditions [6,7]. Biochar was being developed as a good choice of low-cost sorbent with unique properties, especially the modified biochar, e.g., relatively large surface area [8], excellent ion exchange capacity, easy modification to support functional groups, and a range of chemical compositions [9]. Moreover, compared with other sorbents, biochar shows a great affinity for kinds of chromium, due to its surface heterogeneity, abundant porous structure or functional groups [10,11]. In addition, biochar has a negatively charged surface, specific ligands, acidic oxygen groups, and basic nitrogen groups, which is beneficial for the removal of Cr(VI) [12,13,14].

Biochar could be derived from a great variety of waste biomasses or by-products of agriculture, which are relatively inexpensive and more economically and environmentally [15,16,17]. China’s tobacco production ranks first in the world. As reported, the leaf output of Chinese tobacco could achieve 2,325,000 tons in 2014 [18]. Meanwhile, tobacco waste, mainly tobacco stems, reach 3–5 million tons per year [19]. Tobacco stems (TSs) produced abound from the tobacco yard, but they just act as a tobacco residue. The tobacco stem is the major residue of tobacco in the field and causes significant harm to the subsequent crops. Consequently, the convenient way to remove them from the field is by burning or landfills, which also cause serious pollution to the air, soil, and other environmental problems [20]. Thus, it is an environmentally friendly way to obtain biochar from tobacco straw for the sake of treating waste with waste and for the potential use for chromium removal. The modification of the biochar could enhance its metal adsorption capacity through various physical and chemical methods, e.g., surface oxidation, acid activation, and steam activation [21,22]. In addition, biochar is generally prepared in powder, posing a challenge for separation [23]. In this regard, the potential solution is to educe the biochar with a magnetic feature, depending on the magnetic separation characteristic [24]. Additionally, magnetic modification could also add the affinity with Cr anions through chemical adsorption. Ferric complex is regarded as a better choice to modify the biochar for chromium removal [25]. Moreover, the discharge of Cr(III) that results from the reduction in Cr(VI) is another challenge, and amine groups complexed with Cr(III) ions could enhance Cr(III) immobilization [26]. To the author’s knowledge, the biochar derived from tobacco stems with amine groups and modified by Fe_3_O_4_ magnetic particles for chromium removal have rarely been reported.

Biochar derived from tobacco stems (BTSs) was modified with FeCl_3_ magnetic particle and applied for Cr(VI) removal in this study. Tobacco stem biochar could offer binding sites for anion Cr(VI) adsorption, and Fe_3_O_4_ particle modification induced more binding sites. In addition, ferric particles posed magnetic properties to the biochar, which benefit for its separation. The objectives of the present study are (i) to characterize the physicochemical properties of the magnetic biochar (MBTS); (ii) to explore the amine groups that act as the new binding site for Cr(III) removal; and (iii) to assess the potential Cr(VI) removal characteristics and the mechanism by the novel magnetic biochar.

## 2. Materials and Methods

### 2.1. Synthesis of MBTS Magnetic Biochar

A burley tobacco stem was obtained from Hubei Province (China). The TS was air-dried, crushed, and then sieved into 0.25 mm (60 mesh) with an electric pulverize in 30,000 r/min. FeCl_3_·6H_2_O (analytical reagent) together with TS via Fe/TS (m/m) = 0.5 ratio was dissolved in 100 mL deionized water at 100 °C for 6 h, and then dried in an air-cyclic oven (80 °C, 24 h) to acquire the MBTS precursor. The tobacco stem without FeCl_3_·6H_2_O, as well as impregnation with hot water (100 °C) for 6 h (BTS precursor), was used for parallel experiments. The MBTS was pyrolyzed at 400 °C for 30 min, which was raised from 25 °C at a certain rate of 10 °C/min. In the meantime, N_2_ of 99.99% purity was continuously passed through the quartz cylindrical funnel at a rate of 100 mL/min. Then, this was stored in a vacuum (0.1 atm) cooling to room temperature naturally. In order to remove appendiculate impurities, MBTS was washed with ethyl alcohol and distilled water, then dried under 80 °C for 24 h in an air circulated drying oven. Biochar derived from the raw tobacco stem without disposing of FeCl_3_·6H_2_O (BTS) was acquired in the same programmed condition.

### 2.2. Batch Adsorptions

The stock Cr(VI) solution (1 g/L) was preconditioned by dissolving K_2_Cr_2_O_7_ (analytical reagent) into deionized water. All desirable concentrations (30, 40, 50, 60, 70, 80, 90, 100, 150 mg/L) of Cr(VI) solutions were acquired by diluting the prepared stock solution. Then, 20 mg MBTS and BTS was added into 20 mL reaction Cr(VI) solution. Adjust with 0.1 M NaOH and 0.1 M HCl to obtain the desired pH value of the solution. Subsequently, the solution was automatically shaken at 120 rpm under 25, 35, and 45 °C separately. Kinetic experiments were executed at 25 °C, Cr(VI) 30–150 mg/L, and pH = 3.0. After the reaction, the solution was filtered through a 0.22 μm filter, and the collected filtrate was then measured using UV-Vis spectrophotometry (V-5800H, Thermo) at λ = 540 nm. Finally, the adsorption capacity (qt, mg/g) of Cr(VI) can be calculated as the following Equation:

(1) $$\mathrm{qt}=\frac{\left(\mathrm{Co}-\mathrm{Ct}\right)}{m}$$

where C_o_ and C_t_ are the concentration of Cr(VI) at initial and time, t, mg/L; m is the adsorbent dose (g/L). The separated MBTS after adsorption was dried under 80 °C for 6 h in an air-cyclic dried oven for the further characterization.

The adsorption amount experiment of BTS and MBTS at 298 K separately was performed, and the adsorption data were described by the Langmuir model, which then acquired the maximum capacity.

### 2.3. Characterization of MBTS

The surface morphology and pore size of BTS and MBTS were inspected by the Brunauer–Emmet–Teller (BET, Anton Paar, U.S.A., Quadrasorb SI MP 21) specific surface area analyzer, which depended on the adsorption and desorption of nitrogen, using 250 mg BTS or MBTS under 250 °C dried and degassed for 12 h under the N_2_ condition. The micromorphology of MBTS was analyzed with the field emission scanning electron microscope (FESEM/EDS, JEOL JSM-7200F with Oxford Aztec Standard X-max80), with a working voltage of 10.0 kV in the pressure of 1.9 × 10^−4^ Pa. For evaluating the interaction mechanism, X-ray diffraction (XRD, Holland, Philips, PANAnalytical B.V.) patterns were analyzed at a Cu Kα radiation (λ = 1.54178 Å, V = 40 Kv, I = 40 mA), with scanning 2θ from 10° to 80° at 17°/min. The Fourier transform infrared (FTIR, Shimadzu IR Prestige-21) spectrum was taken to analyze the functional groups of BTS and MBTS. Chemical valences of C1s, N1s, O1s, Fe2P, and Cr2P and ion exchange mechanisms were carried out by X-ray photoelectron spectroscopy (XPS, U.K., Kratos AXIS Supra).

### 2.4. Adsorption Kinetics and Isotherms

Adsorption kinetics were applied for simulating the adsorption rate, and the kinetics of the Cr(VI) adsorption was fitted by two universal models as follows:

Pseudo-First-Order Model (PFO Model)

Ln(q_e_ − q_t_) = Lnq_e_ − k_1_t (2)

Pseudo-Second-Order Model (PSO Model)

t/q_t_ = 1/k_2_q_e_^2^ + t/q_e_ (3)

where q_e_ and q_t_ are respective adsorption capacities at equilibrium and time t (mg/g); t is the adsorption time (min); k_1_ and k_2_ are separately first- and second-order rates.

In this study, three adsorption isotherm models were adopted to analyze the adsorption equilibrium data, which are the Langmuir, Freundlich, and Sips isotherms. (Equations (4)–(6)).

(4) $$\mathrm{Langmuir}\;\mathrm{model}:\;q_{e}=\frac{K_{L}q_{\max}C_{e}}{1+K_{L}C_{e}}$$

(5) $$Freundlich\;model:\;q_{e}=\;K_{F}C_{e}^{1/n_{F}}$$

(6) $$\mathrm{Sips}\;\mathrm{isotherms}:\;q_{e}=\frac{q_{\max}{\left(K_{g}C_{e}\right)}^{n_{L}}}{1+{\left(K_{g}C_{e}\right)}^{n_{L}}}$$

where q_e_ represents the Cr(VI) metal ion amount adsorbed at equilibrium, mg/g; Ce represents the equilibrium Cr(VI) concentration, mol/L; q_max_ represents the saturation adsorption capacity, mg/g; K represents the Langmuir constant, L/mg; K_F_ is interrelated to the relative adsorption constant, (mg/g)/(mol/L)^1/n^; K_g_(L/mol) represents the constant of Sips isotherms.; and 1/n_F_ and n_L_ represent the dimensionless exponents of Freundlich and Sips isotherms separately.

## 3. Results and Discussion

### 3.1. Characterization of the Synthesized Biochar

**Figure 1:**
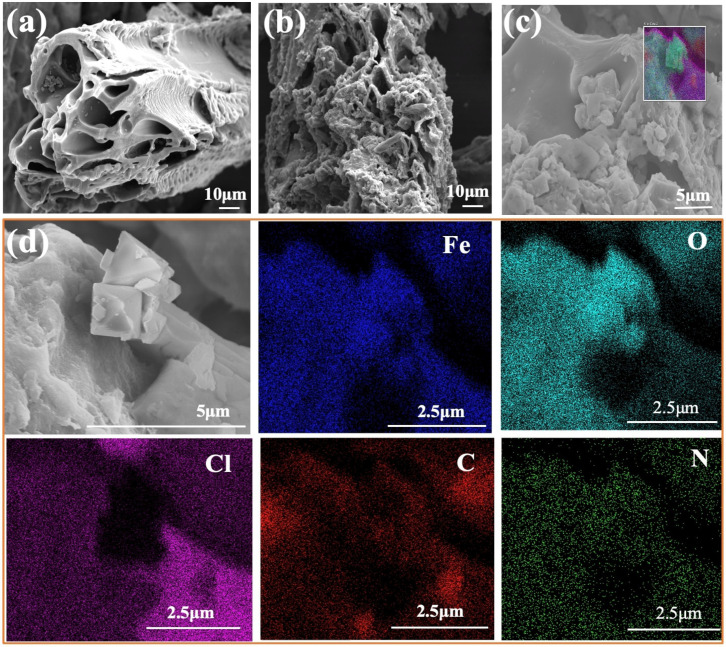
Scanning electron microscopic images. (**a**): BTS; (**b**): MBTS; (**c**): EDS images of MBTS; and (**d**): insert Figure 1c.

The surface morphology and chemical composition of MBTS and BTS were analyzed with SEM-EDS (Figure 1). It was found that that BTS had a relatively smooth sheet shape without the presence of nanoparticles, while MBTS exhibited a rough carbon sheet shape with particles uniformly loaded on its surface (Figure 1b). Figure 1d further reveals a polyhedral structure adhering to the MBTS with the obvious presence of ferric oxides nanoparticles [27]. This was supported by the XRD results showing Fe_3_O_4_ or γ-Fe_2_O_3_ being the main crystalline in MBTS. According to the EDS images (Figure 1d), C, N, O, Fe, and Cl are the four primary elements in MBTS. Furthermore, the crystalline structure adhered to the MBTS, which was mainly constituted with elements of Fe and O, calculating the exist of ferric oxides. 

Table 1 shows the BET analysis of BTS and MBTS. It can be observed that the S_BET_ of MBTS was estimated to be 4.33 m^2^/g, which was significantly smaller than that of BTS (i.e., 32.78 m^2^/g), while the total pore volume of MBTS (i.e., 0.008 cm^3^/g) was also much lower than that of BTS (i.e., 0.072 cm^3^/g). It should also be noted that the average pore radius of both MBTS and BTS were comparable. The pore of BTS was originally large and became smaller as a result of being blocked by ferric after being modified, and then the pores became larger with the dissolving ferric with the acidic solution during the Cr(VI) adsorption experiment. However, unlike from BTS, the analysis reveals that MBTS is highly rich in various functional groups. 

**Figure 2:**
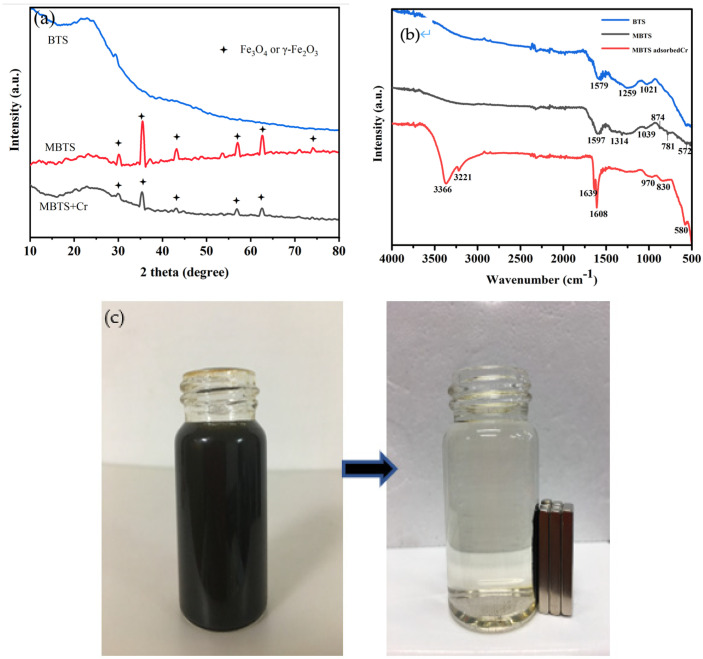
(**a**) The XRD patterns of BTS and MBTS, (**b**) FTIR of MBTS, and (**c**) MBTS dispersed in a water suspension and being magnetically separated.

To further verify the existential form of magnetic iron oxide, the BTS and MBTS materials were characterized by XRD before and after adsorption separately. Figure 2a shows that, except for a slight diffraction peak at 26.7 °C, no obvious peak can be detected for BTS, implying the turbostratic crystallites formatted in BTS [28]. XRD showed that the MBTS and MBTS + Cr may contain Fe_3_O_4_ or γ-Fe_2_O_3_. The formation of iron compounds may pose an important influence on Cr(VI) removal. Furthermore, the after-adsorption pattern (i.e., MBTS + Cr pattern) contains Fe_3_O_4_ or γ-Fe_2_O_3_, demonstrating that the material remains magnetic after adsorption, which is convenient for magnetic separation after adsorption. The surface functional groups of MBTS were analyzed by FTIR. Figure 2b reveals the FTIR data for the BTS, MBTS, and MBTS with adsorbed Cr(VI). The peak at 1039 cm^−1^ and 1314 cm^−1^ was concerning to the C-N group [29,30], and the peak at 1539 cm^−1^ was correlated with the C=C group. Compared with BTS, the new peaks at 781 cm^−1^ and 874 cm^−1^ in FTIR spectra of MBTS, which are in line with the C–H stretching vibration, reveal chemical interactions between MBTS surface and FeCl_3_ [29]. After the biochar was modified with FeCl_3_, a new peak at 572 cm^−1^ conforming with the ferric oxide (Fe_3_O_4_ or γ-Fe_2_O_3_) was presented in the spectra of MBTS, indicating that the tobacco stem biochar was successfully modified with FeCl_3_, which is in accordance with the XRD characterization result. As for the MBTS adsorbed Cr spectra, the peak of nearly 580 cm^−1^ conforms with the representative Fe–O vibration [31], which that after the adsorption of Cr(VI) by the ferric oxides on MBTS, a redox reaction occurred, which reduced Cr(VI) to Cr(III), while the chemical valence of iron changed, then and Fe–O vibration occurred. Figure 2c shows that MBTS disperses in a water suspension and can be magnetically separated.

### 3.2. Adsorption Isotherm and Kinetics

**Figure 3:**
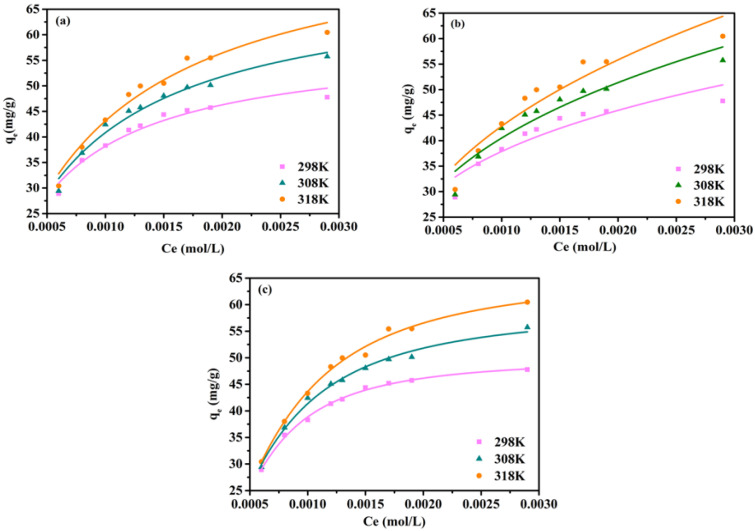
The qt-Ct profiles in the adsorption of Cr(VI) on MBTS at different temperatures. (**a**) Prediction by the Langmuir model; (**b**) Freundlich model; and (**c**) Sips isotherm.

The isotherm data of Cr(VI) adsorption on MBTS were fitted to the Langmuir, Freundlich, and Sips isotherm equations (Figure 3). It appears from Table 2 that the Langmuir and Sips isotherms present a better description for the adsorption data than the Freundlich isotherm. The Sips isotherm can be reasonably simplified to the Langmuir isotherm when the n_L_ value approximates to the unity [32]. The maximum capacity of Cr(VI) adsorption on BTS and MBTS was determined to be 3.84 mg/g and 58.74 mg/g, respectively, at 298 K, which are higher than some reported biochar and magnetic biochar adsorbents listed in Table 3.

Based on the adsorption equilibrium constants determined from the Langmuir isotherm (Table 2), the adsorption thermodynamics were studied by using the following Equations:

ΔG^0^ = −RTlnK_e_ (7)

(8) $${\mathrm{lnK}}_{e}\;=\frac{{△S}^{0}}{R}-\frac{{△H}^{0}}{\mathrm{RT}}$$

where K_e_ means the equilibrium constant of thermodynamic, which approximates the Langmuir equilibrium constant for a diluted solution [36]; ΔG° denominates the change of free energy, kJ/mol; R signifies the gas constant (8.314 J/(mol K)); T expresses the absolute temperature, K; and ΔS° (J/ (mol K)) and ΔH° (kJ/mol) verbalize the change of entropy and enthalpy, separately.

As shown in Table 4, the negative ΔG^0^ evinced the Cr(VI) adsorption on the MBTS was spontaneous, while both the ΔH^0^ value (i.e., 2.842 kJ/mol) and ΔS° value (i.e., 9.903 KJ/mol) were positive, which indicated an endothermic adsorption process [37]. 

**Figure 4:**
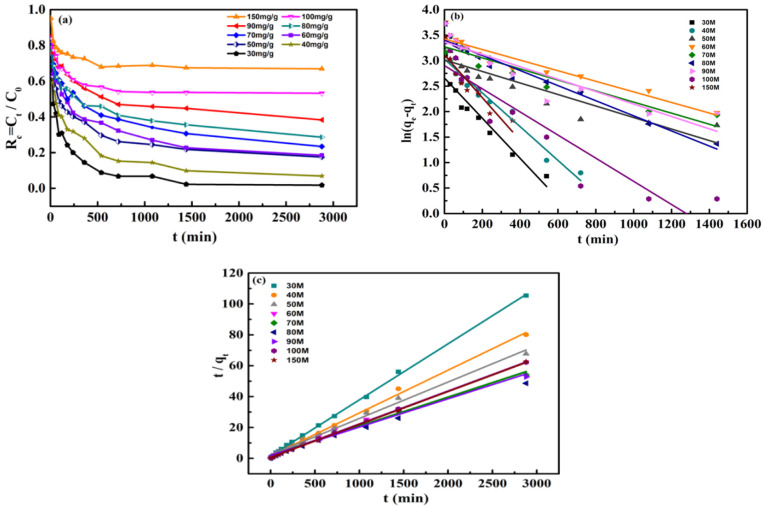
The removal of Cr(VI) on MBTS results for: (**a**) removal efficiencies of different initial concentration versus time; (**b**) PFO linear plots; and (**c**) PSO linear plots. pHCr(VI) = 3.0 ± 0.1, T = 298 ± 1 K.

It appears from Figure 4 that the experimental data could be better depicted by the PSO rate equation. This also showed a chemisorption of Cr(VI) by MBTS. In the light of the above Cr(VI) adsorption behaviors on the MBTS samples, we investigated the contact time to Cr(VI) adsorption on MBTS in diverse Cr(VI) initial concentrations extending from 30 mg/L to 150 mg/L, by using the same MBTS dosage (20 mg/50 mL). The influence of removal time on Cr(VI) adsorption (Figure 4a) illustrates that distinct outset concentrations show identical adsorption laws, and the removal efficiency reduces with the initial enhancement in the Cr(VI) concentration. Figure 4a also reveals the residual Cr(VI) concentration (R_c_ = C_t_/C_0_) with different contact times (t, min). The adsorption rates of Cr(VI) in MBTS were considerably fast at the beginning of the contact time, and no obvious further reduction was measured with the increasing adsorption time after 750 min, inferring that the adsorption of Cr(VI) by MBTS was a chemical process. The kinetics in the adsorption procedure was determined from the curve fitting with the PFO and the PSO. The corresponding detailed kinetic parameters were determined out from the adsorption kinetics. The coefficient value calculated from the PSO model (R^2^ = 0.993) was higher than that of the PFO model (R^2^ = 0.904). The PSO model fitted the adsorption kinetics better between the two kinetic models, demonstrating that the rate-controlling mechanism for the Cr(VI) adsorption on MBTS is chemisorption.

### 3.3. Effect of pH on the Adsorption of Cr(VI) on MBTS

**Figure 5:**
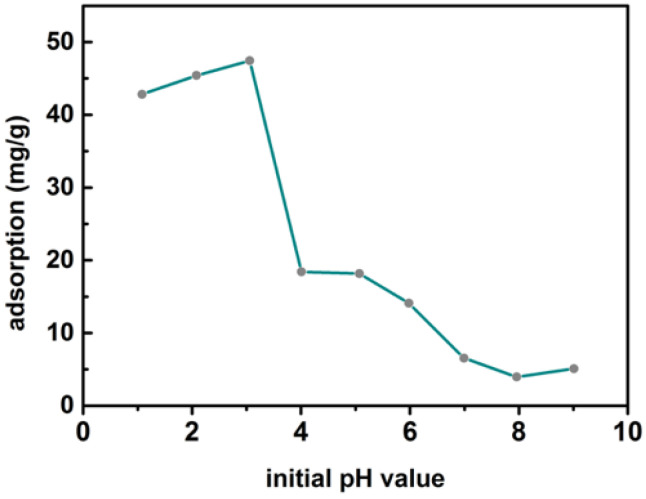
Effect of pH on the Cr(VI) removal.

The pH (1–9) of the solution on the adsorption of Cr(VI) by MBTS was also surveyed under conditions of initial concentration 50 mg/L, dosage 1 g/L, and 24 h contact time. Figure 5 reveals that the Cr(VI) adsorption on MBTS clearly increased at pH = 1.0–3.0 and gradually decreased with the increasing pH, which was consistent with the previous research [38,39]. The maximum adsorption of Cr(VI) on MBTS occurred at a pH of 3. As previously presented in the research, at pH < 2, Cr(VI) mainly existed as Cr_2_$O_{7}^{2-}$, at pH 2–6, both Cr_2_$O_{7}^{2-}$ and ${\mathrm{HCrO}}_{4}^{-}$ were retained, and ${\mathrm{CrO}}_{4}^{2-}$ became dominant when pH < 6 [28,40]. As we know, Cr(VI) was negatively charged and the surface of MBTS was protonated in an acidic environment, showing a positive charge. Therefore, electrostatic attraction was critical in adsorbing the Cr(VI) ion. Whereas, in alkaline or neutral conditions, the surface of MBTS was deprotonated and presented a neutral or negative charge, so the removal efficiency decreased consequently owing to the electrostatic repulsion. 

### 3.4. Effect of Coexisting Ions

**Figure 6:**
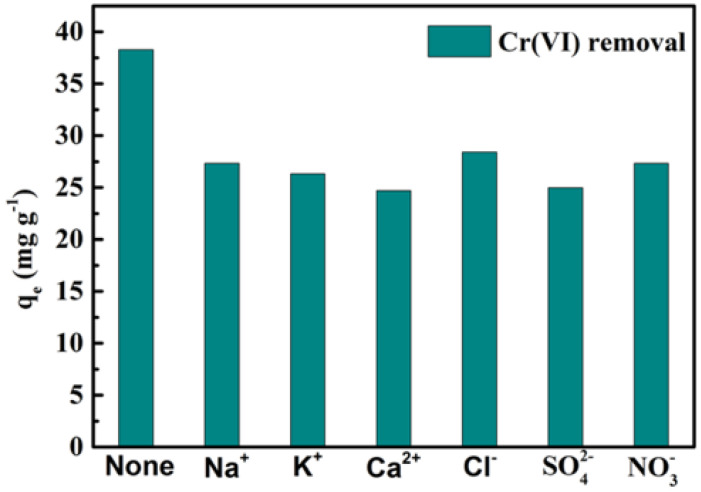
Effect of initial coexisting ion value on the removal of Cr(VI). (I = 0.01 M, pH = 3.0 ± 0.1, T = 298 K).

Moreover, Cr(VI) ions inescapably coexist with diverse electrolyte ions in wastewater, which might power the movement of pollutants. Usually, Na^+^, Ca^2+^, and K^+^ as well as Cl^−^, ${SO}_{4}^{2-}$, and ${NO}_{3}^{-}$ were chosen as the common coexisting ions. Figure 6 describes the impacts of different ions on Cr(VI) (50 mg/L concentration) removal; we found that the coexistence of diverse ions reduced the adsorption ability of Cr(VI) on MBTS. Additionally, the great majority of coexisting ions posed no distinct effect on the Cr(VI) adsorption. In particular, ${SO}_{4}^{2-}$ had more negative impacts on Cr(VI) removal than MBTS, presumably due to ${SO}_{4}^{2-}$ both competing for adsorption sites with Cr(VI) and complexation with the Fe ion [41]. Ca^2+^ in solution was adsorbed on the MBTS and competed with the support sites with Cr(VI), and resulted in fewer adsorbance sites for Cr(VI) [33]. Therefore, it is better to first eliminate the coexisting ${SO}_{4}^{2-}$ and Ca^2+^ to acquire a relatively high removal efficiency of MBTS for Cr(VI) adsorption.

### 3.5. Adsorption Mechanism

**Figure 7:**
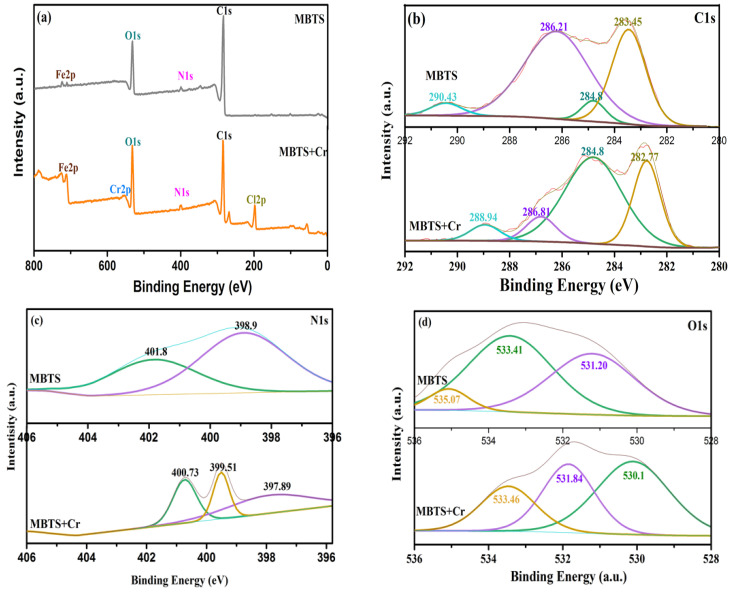

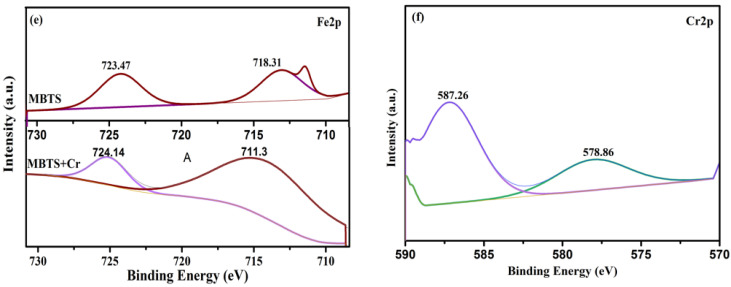
(**a**) XPS spectra; (**b**–**e**) C1s, N1s, O1s, and Fe2p spectra of MBTS before and after adsorption; and (**f**) Cr2p of MBTS.

To investigate the adsorption mechanism between Cr(VI) and MBTS, XPS were supplied with MBTS before and after Cr(VI) adsorption. XPS technology could supply both element analysis and chemical valence changes [42]. As shown in Figure 7a, four major elements are found on MBTS, including C1s, N1s, O1s, and Fe2p. The high-resolution spectrum of Cr2p revealed that Cr element peaks were detected on the after-adsorption spectra, which showed that the chromium adsorbed on the MBTS surface successfully, in comparison with the spectra before adsorption. According to the spectrum of Fe2p (Figure 7e), two characteristic valences of MBTS revealed by XPS result could be peaked at 724.14 eV (Fe2p_1/2_) and 711.3 eV (Fe2p_3/2_) binding energy that was assigned to Fe_3_O_4_ [43], which matched with the XRD results. The resolution of C1s showed four kinds of carbon atoms on MBTS. The peak at 283.45 eV could be assigned to the C–C bond [44], the peak at 284.8 eV was non-oxygenated carbon (C–N bond), the peak at 286.21 eV was a C–O bond, and the peak in 290.43eV was a carboxylate carbon (O–C=O bond) [45], demonstrating that MBTS abounded with oxygen functional groups and C–O binding energy appeared after adsorption. Regarding the O1s, there are three various oxygen-containing functional groups: (a) COOH, 535.07 eV, (b) O-H, 533.41 eV, and (c) C=O, 531.20 eV. It was found that the peak of O–H transformed into 531.84 eV and the intensities of O–the H peaks had a slight decrease after Cr(VI) adsorption. Meanwhile, the peaks at 531.20 eV converted to 530.1 eV, which represented Fe-O functional group [45] becoming significantly higher, meaning that the hydroxyl groups were partially replaced by ${HCrO}_{4}^{-}$/Cr_2_$O_{7}^{2-}$ [46]. The Cr2p spectrum of MBTS showed that Cr2p_3/2_ and Cr2p_1/2_ peaks were situated at 587.26 eV and 578.86 eV, which were characteristic of Cr(VI) and Cr(III). Additionally, the existence of Cr(III) indicated that the Cr(VI) adsorbed on the surface of MBTS, then deoxidated to Cr(III). Figure 7c shows the N1s peaks assigning to the peaks at 398.9 eV for -NH- and 401.8.9 eV for -N=(-C=NH), which were quinoid imine units and benzenoid amine units [47]. After adsorption, a new peak emerged at 400.73 eV for -N=^+^, which should be H^+^ doped on -N=. This is in accordance with the pH increase after adsorption (Figure 5, when the pH of Cr solution < 6), indicating that the protonation promotes the consumption of H^+^. Furthermore, Cr(III) doped on MBTS might be another reason for the production of -NH=^+^. From the above analysis and the experimental results, it can be determined that the removal of Cr(VI) could be dominated by three steps: (1) Cr(VI) exchanged with hydroxyl groups on MBTS; (2) the Cr(VI) adsorbed on MBTS was reduced to Cr(III); and (3) the chelation reaction between Cr(III) and amino groups of MBTS. The responsible groups for the reduction in Cr(VI) to Cr(III) are shown below in Equation (9) to Equation (13) [35,48].

-C-H + Cr(VI) + H_2_O → -C-OH + Cr(III) + H^+^ (9)

-C=O + Cr(VI) + H_2_O → -COOH + Cr(III) + H^+^ (10)

-C-COOH^+^ Cr(VI) + H_2_O → -C-H + Cr(III) + H^+^ + CO_2_↑ (11)

(12) $${\mathrm{Cr}}_{2}O_{7}^{2-}\;+{\mathrm{Fe}}^{2+}(s)\;+\;H_{2}O\;\to \;{\mathrm{Cr}}^{3+}\;+\;{\mathrm{OH}}^{-}\;+\;6{\mathrm{Fe}}^{3+}(s)$$

(13) $$\mathrm{Surface}\;C\;(+)\;+\;{\mathrm{Cr}}_{2}O_{7}^{2-}\;\to \mathrm{Surface}\;C\;-\;{\mathrm{Cr}}_{2}O_{7}\;(\mathrm{Surface})$$

### 3.6. Engineering Implication

**Figure 8:**
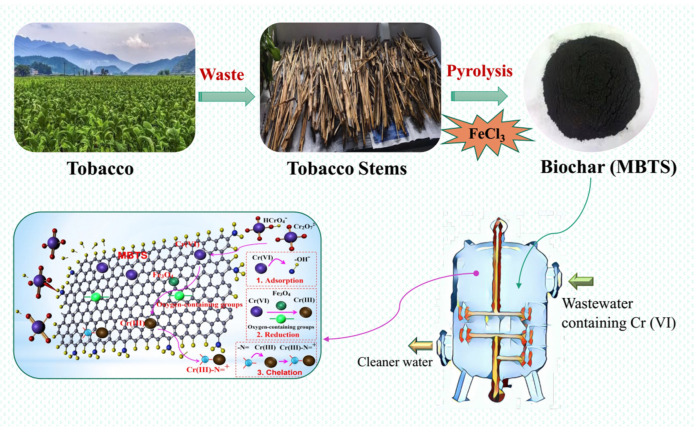
The engineering implication and mechanism of MBTS on Cr(VI) removal.

As the main residue of tobacco cultivation, the treatment focused on incineration, landfill, or allowed for decomposition [49], which poses a great burden and pollution to the environment. Additionally, the tobacco stems were added to stoves for heating or cooking but had just a 10% burning efficiency [50]. Tobacco stems are a precious and valuable resource. Some studies have shown that biochar derived from tobacco stems had a better effect in treating heavy metals in wastewater and improving soil amendment [51,52,53]. The preparation of tobacco stems into biochar enhanced its own added value and could be applied in the industry. However, to the best of the author’s knowledge, this was the first time that the modified biochar made from tobacco stems was found to have a high adsorption activity for hexavalent chromium. Through modification, the Cr(VI) adsorption capacity evidently improved, reaching a 15 time higher capacity than the raw tobacco stem biochar in this study. Therefore, biochar derived from tobacco stems modified by Fe_3_O_4_ magnetic particles could be used for the treatment of chromium-contaminated wastewater, thus achieving the purpose of waste disposal and becoming more economical (Figure 8). This concept was in accordance with the circular economy conception for potential metal-polluted wastewater treatment. 

## 4. Conclusions

In this work, new magnetic biochar originated from a burley tobacco stem (MBTS) was fit for Cr(VI) removal. The optimization conditions for the sample preparation were pyrolysis at 400 °C for 1h and a mass ratio of Fe: MBTS of 0.5. Through the modification of ferric ion, MBTS contained abundant function groups (C–H, C=N, and C=O). Both the pH and coexisted ions had an important influence on the removal of Cr(VI). The Cr(VI) removal mechanism followed by three steps: Cr(VI) was reduced to Cr(III), after it was adsorbed and exchanged OH with MBTS; then, Cr(III) produced a complex reaction with the amino groups of MBTS. The novel biochar material showed an outstanding Cr(VI) removal capacity, and the maximum adsorption capacity of MBTS was 58.74 mg/g, much higher than burley tobacco stem biochar (BTS). Therefore, the MBTS prepared in this study has a prospective application for removing Cr(VI) from contaminated water.

## Figures and Tables

**Table 1:** BET characteristics of modified and pristine biochar.

Adsorbent	BET Surface Area (m^2^/g)	Total Pore Volume (cm^3^/g)	Average Pore Radius (nm)
BTS	32.78	0.072	1.631
MBTS before adsorption	4.33	0.008	1.633
MBTS after adsorption	13.83	0.039	1.637

**Table 2:** Adsorption isotherms of Cr(VI) on MBTS.

	T(K)	q_m_ (mg/g)	K_L_ (L/mol)	K_f_ ((mg/g)/ (mol/L)^1/n^)	Kg (L/mol)	R^2^	1/n_F_	n_L_
Langmuir model	298	58.74	1856.77			0.959		
	308	70.89	1358.20			0.973		
	318	70.89	1121.19			0.970		
Freundlich model	298			258.66		0.860	0.28	
	308			433.26		0.903	0.34	
	318			602.06		0.900	0.38	
Sips isotherm	298	49.83			1998.19	0.996		1.83
	308	59.24			1692.24	0.990		1.59
	318	65.97			1512.75	0.991		1.62

**Table 3:** Comparisons of adsorption capacities of Cr(VI) ions on biochar and magnetic biochar with reported adsorbents.

Absorbent	Modified Method	q_m_ (mg/g)	Reference
BTS	Biochar derived from tobacco stems	3.84	This study
MBTS	BTS modified with FeCl_3_	54.92	This study
PBC-ND	Biochar derived from bamboo and poplar	5.4	[33]
Fe/PBC-ND	PBC-ND modified with Fe (NO_3_)_3_	25.68	[33]
BM-Fe-HC	Biochar modified with FeCl_3_	48.1	[34]
PC	Porous carbon	2.50	[35]
Fe@PC	PC modified with Fe (NO_3_)_3_	10.07	[35]
Magnetic biochar	Biochar modified with FeCl3	27.2	[28]

**Table 4:** Thermodynamics parameters for Cr(VI) adsorption.

Adsorbent	ΔH^0^ (KJ/mol)	ΔS^0^ (J/mol K)	ΔG^0^ (KJ/mol)		
			298 K	308 K	318 K
MBTS	2.842	9.903	−0.109	−0.208	−0.307

## Data Availability

Not applicable.
